# Prognostic Value of Survival of MicroRNAs Signatures in Non-small Cell Lung Cancer

**DOI:** 10.7150/jca.30336

**Published:** 2019-10-06

**Authors:** Bo Chen, Tianshun Gao, Weiwei Yuan, Weihong Zhao, Tza-Huei Wang, Jianqing Wu

**Affiliations:** 1 Jiangsu Provincial Key Laboratory of Geriatrics, Department of Geriatrics, the First Affiliated Hospital with Nanjing Medical University, Nanjing, Jiangsu 210029, China.; 2 Department of Biomedical Engineering, Johns Hopkins University School of Medicine, Baltimore, Maryland 21205, USA.; 3 Wilmer Bioinformatics, Johns Hopkins Hospital, Baltimore, Maryland 21231, USA.; 4 Department of Mechanical Engineering, Johns Hopkins University, Baltimore, Maryland 21218, USA.; 5 The Sidney Kimmel Comprehensive Cancer Center, Johns Hopkins University School of Medicine, Baltimore, Maryland 21287, USA.; 6 Johns Hopkins Institute for NanoBioTechnology, Johns Hopkins University, Baltimore, Maryland 21218, USA.

**Keywords:** MicroRNAs, Non-small cell lung cancer, Survival analysis, Prognostic value, Functional enrichment

## Abstract

**Introduction:** Accumulating evidence showed that a large number of microRNAs (miRNAs) are abnormally expressed in lung cancer tissues and play critical roles in cancer development and progression. The aim of this study is to identify the differentially expressed miRNAs (DEMs) between non-small cell lung cancer (NSCLC) and normal lung tissues, and evaluate the prognostic value and potential target gene functional enrichment of the DEMs. **Materials and Methods:** We first downloaded the high-throughput miRNA data from The Cancer Genome Atlas Project (TCGA) database, and subsequently analyzed the data using bioinformatics analysis including limma package in R, Kaplan-Meier curve and Log-rank method, and several online analysis tools. **Results:** A total of 125 DEMs and 138 DEMs were respectively identified in lung adenocarcinoma (LUAD) tissues and lung squamous cell carcinoma (LUSC) tissues compared with their matched normal tissues. Moreover, we found that the prognostic function of the eight miRNAs (miR-375, miR-148a, miR-29b-1 and miR-584 for LUAD; miR-4746, miR-326, miR-93 and miR-671 for LUSC). Furthermore, the two four-miRNA signatures were constructed and found to be an independent prognostic factor for LUAD and LUSC patients, respectively. Additionally, our results indicated that the target genes of eight miRNAs may be involved in various pathways related to NSCLC, including PI3K-Akt, TGF-beta, FoxO, Ras, GPI-anchor biosynthesis and metabolic, Rap1, HIF-1 and proteasome. **Conclusion:** Overall, eight miRNAs were closely correlated with survival of NSCLC patients, and the constructed two four-miRNA signatures could be respectively used as prognostic markers in LUAD and LUSC patients.

## Introduction

Lung cancer is the leading cause of cancer deaths worldwide because of its high incidence and associated mortality 1. Non-small cell lung cancer (NSCLC) accounts for over 80% of all lung carcinomas and continues to increase in incidence 2, with a 5-year survival rate of only 10%. The treatments are often less effective in advanced stages compared with early interventions. Thus, it remains of great importance to identify novel biomarkers and explore potential molecular mechanisms of NSCLC for early detection and treatments of NSCLC.

MicroRNAs (miRNAs), a key component of the noncoding RNA family, are approximately 19-25 nucleotides that involved in the post-transcriptional regulation of gene expression. It has been shown that miRNAs are aberrantly expressed in different types of cancer including lung cancer and function either as oncogenes or tumor suppressors 3, 4. Multiple evidence has demonstrated that miRNAs regulated various tumorigenesis processes including cell maturation, proliferation, apoptosis, motility, invasiveness, and autophagy 5-7. Thus, miRNAs have a great potential to serve as promising markers in the diagnosis, prognosis, and personalized targeted therapies.

Although a number of miRNAs have been identified in predicting the clinical outcome in lung cancer, there exists inconsistence in previous studies 8. This may due to the small sample size, heterogeneous histological subtype, different detection platforms, and various data processing methods. The Cancer Genome Atlas Project (TCGA) is a National Cancer Institute effort to profile more than 20 different tumor types using genomic platforms and to make raw and processed data available to all researchers. The TCGA released a large number of miRNA sequencing data for lung cancer patients. The aim of this study was to identify the differentially expressed miRNAs (DEMs) between NSCLC tissues and matched normal lung tissues by analyzing the high-throughput miRNA data downloaded from TCGA database. Additionally, we evaluated the prognostic value of the DEMs and constructed two four-miRNA signatures that could effectively predict patient survival. Furthermore, we analyzed the pathway and function of the target genes of selected prognostic miRNAs, which may provide novel insights into understanding the underlying molecular mechanism of NSCLC.

## Materials and methods

### Data processing

The raw sequencing data and clinical information were downloaded from TCGA database (https://cancergenome.nih.gov/). The inclusion criteria were set as follows: 1 the sample with both miRNA sequencing data and clinical information; 2 the sample with prognosis information. Finally, a total of 567 samples and 523 samples were separately enrolled in this study, including 513 lung adenocarcinoma (LUAD) tissues and 54 matched normal tissues, and 478 lung squamous cell carcinoma (LUSC) tissues and 45 matched normal tissues. The detailed clinical characteristics and data of expressed miRNAs were list in Table S1 and S2. The miRNA sequencing data were processed using R language package. The DEMs between NSCLC and normal tissues were analyzed by limma package in R. The fold changes (FCs) in the expression of individual miRNA were calculated and DEMs with |log2FC| > 1.0 and P < 0.05 were considered to be significant.

### Association of DEMs and patient prognosis

The DEMs profiles were normalized by log2 transformed. The prognostic value of each DEM was evaluated using Kaplan-Meier curve and Log-rank method. The miRNAs that were significantly associated with overall survival (OS) were identified as prognostic miRNAs, and then subjected to a binary logistic regression analysis. Specifically, we used the “Surv”, “survfit” and “ggsurvplot” functions in R package “survminer” to evaluate each miRNA's performance and decide if it can be taken as the prognostic signature by its p-value. For each miRNA, we firstly calculated its median value of all expression scores and set it as the cutoff to classify all expression scores into “high” and “low” groups. Then a survival object was set by the correlation between the survival time and survival status. Finally, we created a survival curve to fit the relationship between classified miRNA group and the survival object by the Kaplan-Meier formula. A p-value was also calculated in this process and would tell if this miRNA is a prognostic signature. Subsequently, two prognostic four-miRNA signatures were constructed, and the miRNA signatures could calculate a risk score for each NSCLC patient. With the two prognostic four-miRNA signatures, LUAD and LUSC patients were classified into high risk and low risk groups using the median risk score. Then, the differences in patients' survival between the high risk group and low risk group were evaluated by Kaplan-Meier method.

### The target gene prediction of prognostic miRNAs

The target genes of prognostic miRNAs were predicted using MicroT-CDS (http://diana.imis.athena-innovation.gr/DianaTools/index.php?r=microT_CDS), miRDB (http://www.mirdb.org/miRDB/), miRTarBase (http://mirtarbase.mbc.nctu.edu.tw/), and TargetScan (http://www.targetscan.org/) online analysis tools. To further enhance the bioinformatics analysis reliability, the overlapping target genes were identified using Venn diagram. Then, the overlapping genes were analyzed by The Database for Annotation, Visualization and Integrated Discovery (DAVID) bioinformatics tool (https://david.ncifcrf.gov/). DAVID is a web-based online bioinformatics resource that aims to provide a comprehensive set of functional annotation tools for the investigators to understand the biological mechanisms associated with large lists of genes/proteins. Gene Ontology (GO) and Kyoto Encyclopedia of Genes and Genomes (KEGG) pathway enrichment analyses were then performed for the target genes. The *P*-value < 0.05 and gene count ≥ 3 were set as the cut-off criteria.

### Construction of protein-protein interaction (PPI) network

The PPI network of aforementioned overlapping target genes was constructed using the Search Tool for the Retrieval of Interacting Gene (STRING; http://string-db.org/) database. STRING is an online tool to predict the protein-protein interaction information, and can provide a system-wide view of cellular processes. To evaluate the interactive relationships among target genes, we established the PPI network using STRING, and “Confidence score > 0.4” was selected as the cut-off criterion. Then, PPI network was visualized by Cytoscape software (http://www.cytoscape.org/). The hub proteins are a small number of proteins that have many interactions with other proteins.

### Statistical analysis

The data were expressed as mean ± standard deviation (SD). The expression levels of miRNAs in lung cancer and matched normal tissues were analyzed by unpaired *t-*test. The chi-square and *t*-tests were performed to assess the relationship between miRNAs expression and clinical features. Kaplan-Meier survival analysis and univariate/multivariate Cox proportional hazard regression analysis were carried out to compare each miRNA (low vs. high level). *P* value less than 0.05 was considered as statistical significant. The statistical analysis was performed using IBM SPSS Statistics software program version 22.0 (IBM Corp., NY, USA).

## Results

### Identification of DEMs in NSCLC

**Figure 1:**
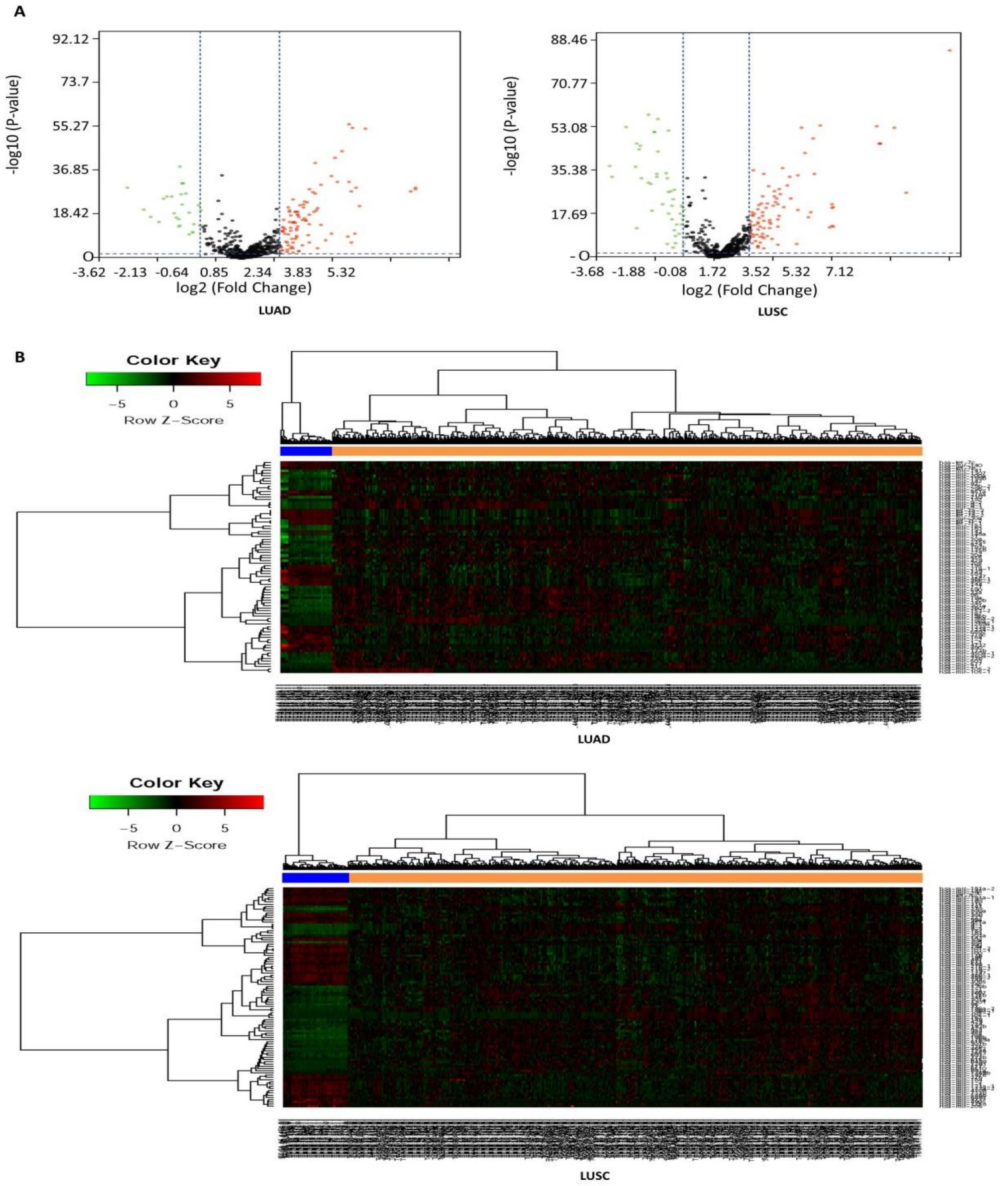
Volcano plot and hierarchical clustering of differentially expressed miRNAs (DEMs). (A) Volcano plot of DEMs between NSCLC tissues and matched normal tissues. The red dot represents up-regulated miRNA, and green dot represents down-regulated miRNA. (B) Hierarchical clustering of DEMs between NSCLC tissues and matched normal tissues. The orange column represents cancer tissues, and blue column represents matched normal tissues.

In this study, a total of 567 samples and 523 samples were separately enrolled in this study, including 513 LUAD tissues and 54 matched normal tissues, and 478 LUSC tissues and 45 matched normal tissues. The detailed clinical characteristics include age at diagnosis, T stage, lymph node status, metastasis, stage, and smoking history category (Table 1). According to the cut-off criteria (*P* < 0.05 and |log2FC| > 1.0), a total of 125 DEMs were identified between LUAD tissues and matched normal tissues, including 95 up-regulated and 30 down-regulated miRNAs (Table S3), and a total of 138 DEMs were identified between LUSC tissues and matched normal tissues, including 89 up-regulated and 49 down-regulated miRNAs (Table S4). In order to prove the *P* value and |log2FC| whether conform to logic with a different test, we present the result as volcano plot (Fig. 1A). The hierarchical clustering of DEMs was performed using the heatmap.2 function of the gplots package in Various R Programming tool (version 2.12), and the results indicated that NSCLC tissues could be distinguished from matched normal tissues (Figure 1B).

### Identification of eight miRNAs associated with overall survival (OS) in NSCLC

**Figure 2:**
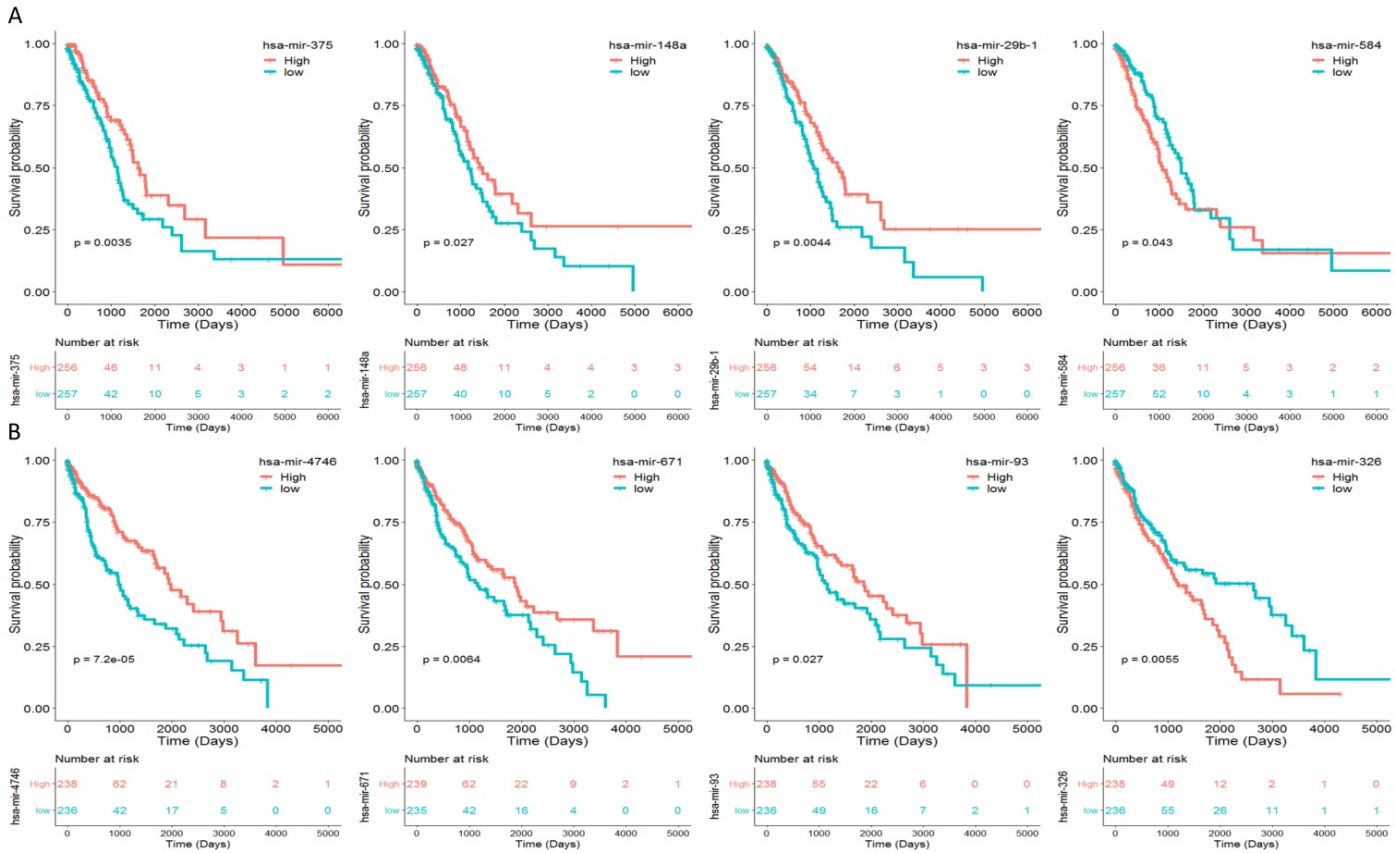
Eight miRNAs were associated with overall survival (OS) in NSCLC patients by using Kaplan-Meier curve and Log-rank test. The patients were stratified into high level group and low level group according to the median of each miRNA. (A) The results of LUAD patients; (B) The results of LUSC patients.

To identify the miRNAs which would be potentially associated with OS of NSCLC patients, we evaluated the association between miRNAs expression and patients' survival using Kaplan-Meier curve and Log-rank test. The results showed that in LUAD samples, three miRNAs (miR-375, miR-148a and miR-29b-1) were positively correlated with OS, and one miRNA (miR-584) was negatively related to OS (Fig. 2A). In LUSC samples, three miRNAs (miR-4746, miR-93 and miR-671) were positively correlated with OS, and one miRNAs (miR-326) was negatively related to OS (Fig. 2B). The association between aforementioned miRNAs and clinical features was evaluated in NSCLC patients (Table 2). The results showed that in LUAD patients, miR-148a was significantly associated with smoking history category (*P* = 0.018); miR-29b-1 was significantly associated with lymph node status (*P* = 0.016) and stage (*P* = 0.011); miR-584 was significantly associated with T stage (*P* = 0.008) and stage (*P* = 0.026). No significant difference was found between miR-375 and their clinical features (*P* > 0.05). As for LUSC patients, miR-4746 was significantly associated with age at diagnosis (*P*<0.001); miR-93 was significantly associated with diagnosis age (*P*=0.001), metastasis (*P*=0.031) and smoking history category (*P*=0.018); miR-671 was only significantly associated with diagnosis age (*P*<0.001). Additionally, we did not found any significant difference between miR-326 and clinical features (*P* > 0.05).

### Prognostic value of eight miRNAs signature risk score in NSCLC

**Figure 3:**
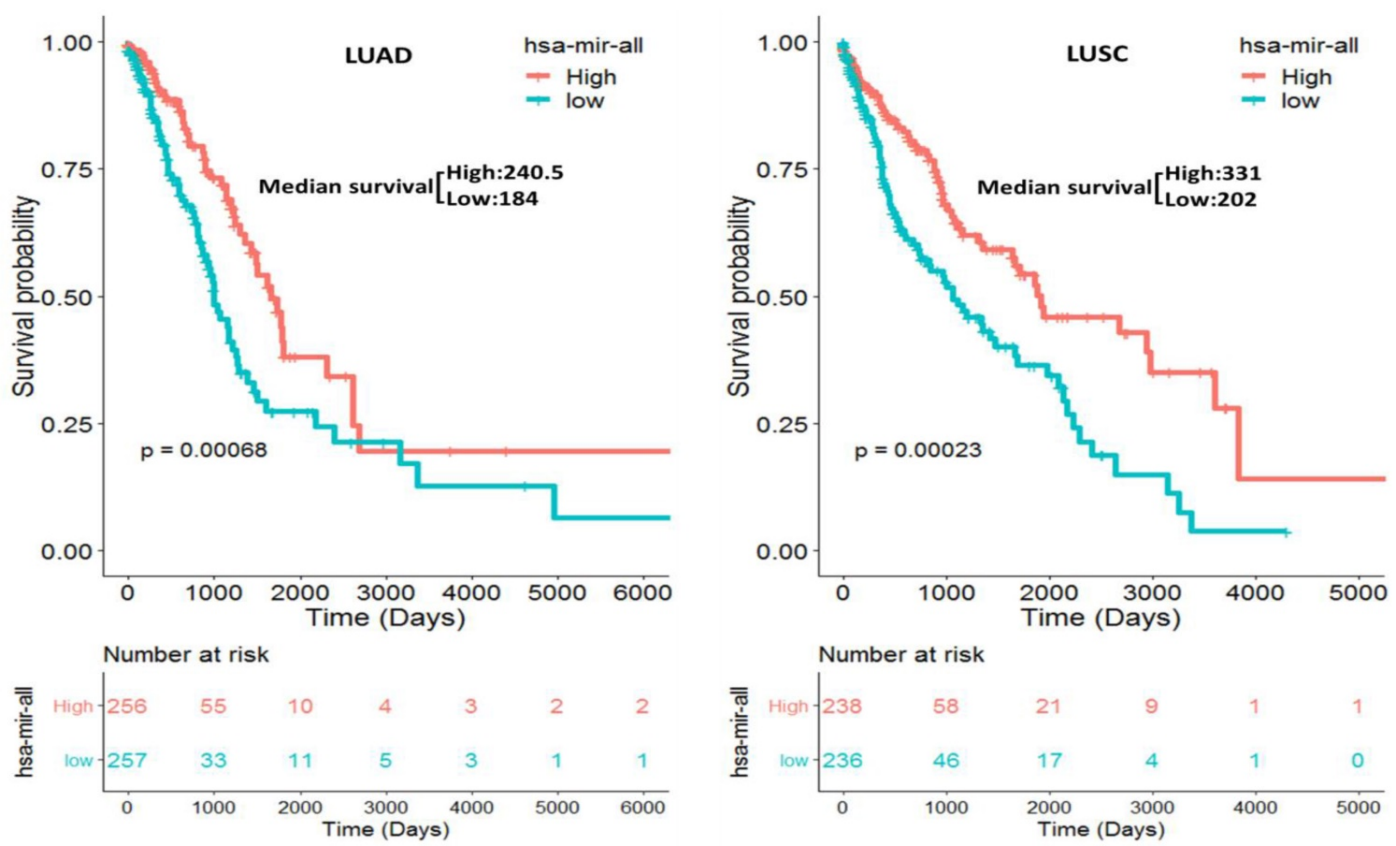
Kaplan-Meier curve for the two constructed prognostic four-miRNA signatures in NSCLC (LUAD: miR-375, miR-148a, miR-29b-1 and miR-584; LUSC: miR-4746, miR-326, miR-93 and miR-671) patients. The patients were stratified into high risk group and low risk group based on the median.

We separately constructed two prognostic miRNA signatures (four-miRNA signature for LUAD: miR-375, miR-148a, miR-29b-1 and miR-584; four-miRNA signature for LUSC: miR-4746, miR-326, miR-93 and miR-671) by integrating the expression profiles of two four-miRNA and corresponding estimated regression coefficient. Then, we calculated a risk score for each patient and ranked them according to increased score. Thus, a total of 513 LUAD patients were classified into a high risk group (n = 256) and a low risk group (n = 257) according to the median risk score. Meanwhile, a total of 474 LUSC patients were also classified into a high risk group (n = 238) and a low risk group (n = 236) according to the median risk score. Survival analysis was performed using the Kaplan-Meier method with a Log-rank statistical test. The results showed that patients in high risk group have significantly worse OS than patients in low risk group (*P* = 6.80E-04 for LUAD, *P* = 2.30E-04 for LUSC, Fig. 3) both in LUAD and LUSC patients.

Taking into account the following clinical features: age, metastasis, lymph node status, stage, T stage, and smoking history category, univariate and multivariate Cox regression analysis were used to test the effect of the two constructed prognostic four-miRNA signatures (high risk vs. low risk) on OS. In univariate analysis, lymph node status (HR = 2.58, *P* = 9.22E-08), stage (HR = 2.90, *P* = 2.54E-09), T stage (HR = 2.47, *P* = 1.73E-04), and four-miRNA signature (HR = 1.85, *P* = 6.76E-04) were associated with OS in LUAD patients. In multivariate analysis, the four-miRNA signature (HR = 1.78, *P* = 9.66E-03) was showed to be an independent prognostic factor in LUAD patients (Table 3). Meanwhile, in univariate analysis, stage (HR = 1.54, *P* = 2.89E-02), T stage (HR = 1.53,* P* = 2.49E-02), and four-miRNA signature (HR = 1.85, *P* = 2.32E-04) were associated with OS in LUSC patients. In multivariate analysis, the four-miRNA signature (HR = 2.44, *P* = 5.17E-06) was also showed to be an independent prognostic factor in LUSC patients (Table 3).

### Target prediction, PPI network and function analysis

**Figure 4:**
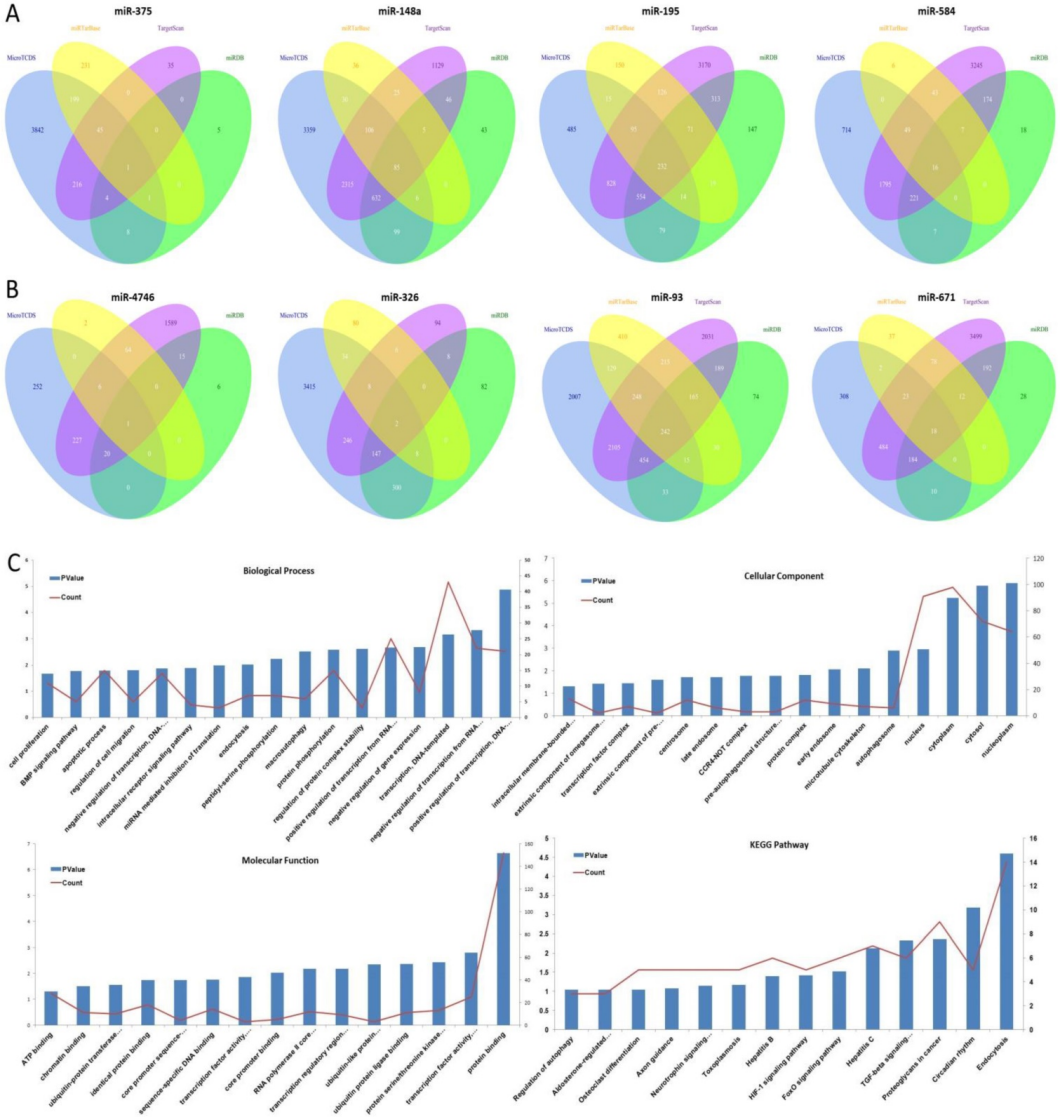
The target gene prediction and function analysis. The overlapping target genes were predicted using MicroT-CDS, miRDB, miRTarBase, and TargetScan online analysis tools. (A) Venn diagram of the overlapping target genes in LUAD patients; (B) Venn diagram of the overlapping target genes in LUSC patients; (C) The significantly enriched biological process, cellular component, molecular function, and KEGG pathways of miR-93 target genes.

The target genes of eight aforementioned prognostic miRNAs were predicted using MicroT-CDS, miRDB, miRTarBase, and TargetScan online analysis tools. A total of 1 overlapping gene of miR-375, 85 overlapping genes of miR-148a, 5 overlapping genes of miR-29b-1, and 16 overlapping genes of miR-584 were identified in LUAD samples (Fig. 4A). Meanwhile, a total of 1 overlapping gene of miR-4746, 2 overlapping genes of miR-326, 242 overlapping genes of miR-93, and 18 overlapping genes of miR-671 were identified in LUSC samples (Fig. 4B). Then, enrichment analysis was performed to elucidate the biological function of consensus target genes.

As for LUSC miR-93, the GO biological process (BP) terms, cellular component (CC), molecular function (MF), and KEGG pathways were respectively analyzed using DAVID bioinformatics tool as mentioned above, and the results are as follows (Fig. 4C). In BP, the genes were mainly enriched in regulation of transcription, regulation of protein complex stability and phosphorylation, regulation of macroautophagy and endocytosis, regulation of intracellular receptor signaling pathway, and regulation of cell migration, proliferation and apoptosis. In CC, these genes were mainly enriched in nucleoplasm, cytosol, cytoplasm, nucleus, autophagosome, microtubule cytoskeleton, early endosome, and protein complex. In MF, they were mainly associated with protein binding, transcription factor activity, sequence-specific DNA binding, protein serine/threonine kinase activity, ubiquitin protein ligase binding, ubiquitin-like protein transferase activity, and transcription regulatory region DNA binding. The KEGG pathways were significantly enriched in endocytosis, circadian rhythm, proteoglycans in cancer, TGF-beta signaling pathway, FoxO signaling pathway, HIF-1 signaling pathway, and hepatitis.

**Figure 5:**
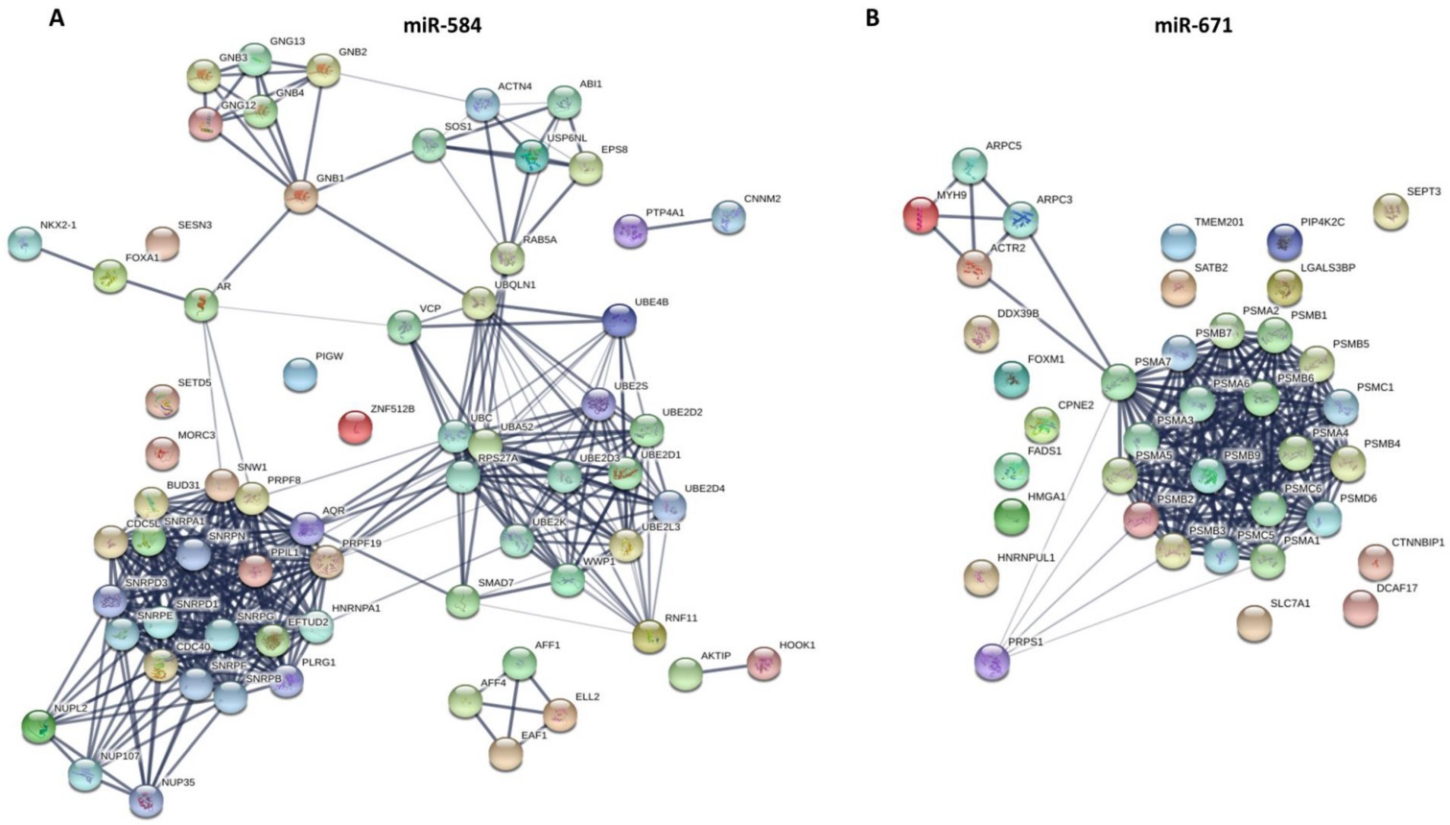
The PPI networks of two miRNAs overlapping target genes were constructed using the Search Tool for the Retrieval of Interacting Gene (STRING; http://string-db.org/) database. (A) miR-584; (B) miR-671.

Other aforementioned predicted overlapping target genes of the prognostic miRNAs were respectively submitted to the STRING database to predict the protein-protein interactions and potential function, and the most significant modules were screened out. As for miR-375, the BP was mainly enriched in SRP-dependent cotranslational protein targeting to membrane, nuclear-transcribed mRNA catabolic process, nonsense-mediated decay, and mRNA metabolic process. The CC and KEGG pathway were mainly enriched in the ribosome. The MF was mainly enriched in the structural constituent of ribosome and RNA binding. As for miR-148a, the BP was mainly enriched in toxin transport, binding of sperm to zona pellucida, and regulation of the apoptotic process. The CC was mainly enriched in chaperonin-containing T-complex, zona pellucida receptor complex, cytosol, micro-ribonucleoprotein complex, and RNAi effector complex. The MF was mainly enriched in protein binding, pre-miRNA binding, nucleotide binding, and purine ribonucleoside binding. The KEGG pathways were mainly enriched in miRNAs in cancer, PI3K-Akt signaling pathway, and other pathways in cancer. As for miR-29b-1, the BP was mainly enriched in muscle filament sliding, actin filament-based movement, and S-adenosylmethionine metabolic process. The CC was mainly enriched in myofibril, sarcomere and cytosol. The MF was mainly enriched in actin binding, cytoskeletal protein binding, and structural constituent of muscle. The KEGG pathways were mainly enriched in cysteine and methionine metabolism, and cardiac muscle contraction. As for miR-584 (Fig. 5), the BP was mainly enriched in mRNA splicing via spliceosome, cellular response to glucagon stimulus, and energy reserve metabolic process. The CC was mainly enriched in catalytic step 2 spliceosome, spliceosomal complex, and nucleus. The MF was mainly enriched in enzyme binding and GTPase binding. The KEGG pathways were mainly enriched in spliceosome, Ras signaling pathway, and circadian entrainment. As for miR-4746, the BP were mainly enriched in glycosylphosphatidylinositol (GPI)-anchor biosynthetic process, phosphatidylinositol biosynthetic process, C-terminal protein lipidation, lipoprotein biosynthetic process, and preassembly of GPI anchor in ER membrane. The CC was mainly enriched in glycosylphosphatidylinositol-N-acetylglucosaminyltransferase (GPI-GnT) complex, endoplasmic reticulum membrane, nuclear outer membrane-endoplasmic reticulum membrane network, and endoplasmic reticulum. The MF was mainly enriched in phosphatidylinositol N-acetylglucosaminyltransferase activity, transferase activity, transferring hexosyl groups, and phosphotransferase activity for other substituted phosphate groups. The KEGG pathways were mainly enriched in GPI-anchor biosynthesis and metabolic pathways.

As for miR-326, the BP was mainly enriched in the ephrin receptor signaling pathway, axon guidance, axonogenesis, and axon development. The CC was mainly enriched in the plasma membrane, cell periphery, and the anchored component of the membrane. The MF was mainly enriched in ephrin receptor binding and activity, receptor binding, and transmembrane-ephrin receptor activity. The KEGG pathways were mainly enriched in axon guidance, Ras signaling pathway, and Rap1 signaling pathway. As for miR-671 (Fig. 5), the BP was mainly enriched in antigen processing and presentation of exogenous peptide antigen via MHC class I (TAP-dependent), regulation of cellular amino acid metabolic process, DNA damage response, and signal transduction by the p53 class mediator. The CC was mainly enriched in proteasome complex, proteasome core complex and alpha-subunit complex, cytosol, extracellular exosome, and nuclear part. The MF was mainly enriched in threonine-type endopeptidase activity, hydrolase activity, and proteasome-activating ATPase activity. The KEGG pathways were mainly enriched in regulation of proteasome.

## Discussion

MiRNAs are highly conserved noncoding RNAs of about 19-25 nucleotides. Through specifically pairing with complementary sites in 3' untranslated regions (UTRs) of target mRNAs, they mediate post-transcriptional silencing. MiRNAs have been implicated in many physiological processes including proliferation, differentiation, development, apoptosis, and metabolism. In recent years many studies have revealed that the aberrant expression of miRNA is closely related to tumorigenesis and is now an intense field of study. Lung cancer is the most common cause of death from cancer worldwide, patients with advanced stage of lung cancer, have a median survival time of only 10 months. Lung cancer can be separated into two major forms: non-small cell lung cancer (NSCLC) and small cell lung cancer (SCLC), which account for 80 and 20% of all lung carcinomas, respectively 9. The NSCLC patient prognosis would be improved considerably if tumor behavior could be predicted reliably at the time of initial diagnosis. Therefore, understanding the molecular mechanisms of lung cancer development and identification of novel biomarkers are needed. In this study, a total of 125 and 138 DEMs were identified in LUAD and LUSC patients respectively, and eight of them were associated with OS in NSCLC patients. Notably, the two four-miRNA signatures (miR-375, miR-148a, miR-29b-1 and miR-584 for LUAD; miR-4746, miR-326, miR-93 and miR-671 for LUSC) were constructed and found to be an independent prognostic factor for NSCLC patients. Moreover, we screened the target genes of these eight miRNAs, and predicted the enrichment gene ontology and signal pathways of target genes using bioinformatics methods, including biological process, cellular component, molecular function and KEGG pathway.

MiRNAs, as the master modulators of multiple biological and pathological processes, are a hot research topic in the field of cancer development and progression. Multiple evidence has demonstrated that miRNAs established a complex combinatorial system of gene expression and pathway regulation, as well as prognostic indicators and therapeutic targets in various cancers, including lung cancer. Previous studies have demonstrated that many miRNAs are crucial for the initiation, progression and metastasis of lung cancer by regulating various processes, including cancer initiation and progression. To date, several studies had identified a number of miRNAs with prognostic values in NSCLC, such as miR-21, miR-200c, miR-125b, miR-148b, miR-365, miR-124, miR-32, miR-146a, and so on 10. However, previous studies were based on small sample size, sample types, different detection platforms, various assay methods, and relatively limited numbers of miRNAs. In the present study, we analyzed high-throughput data, and identified that three miRNAs (miR-375, miR-148a and miR-29b-1) were positively and one miRNA (miR-584) was negatively associated with clinical outcome of LUAD patients. Meanwhile, three miRNAs (miR-4746, miR-93 and miR-671) were positively and one miRNA (miR-326) was negatively associated with clinical outcome of LUSC patients. MiR-375 was first identified as a pancreatic islet-specific miRNA regulating insulin secretion. However, further study revealed that miR-375 is a multifunctional miRNA participating in pancreatic islet development, glucose homeostasis, mucosal immunity, lung surfactant secretion and more importantly, tumorigenesis. Recently, miR-375 has been found significantly downregulated in multiple types of cancer, and suppresses core hallmarks of cancer by targeting several important oncogenes like AEG-1, YAP1, IGF1R and PDK1. The alteration of miR-375 in cancer is caused by a variety of mechanisms, including the dysregulation of transcription factors, aberrant promoter methylation and so on. Reduced expression of miR-375 in tissue or circulation may indicate the presence of neoplasia as well as a poor prognosis of many malignant cancers 11. MiR-148a is aberrantly expressed in various cancers and has been identified as an oncogenic or tumor suppressor with crucial roles in the molecular mechanisms of oncogenesis. The role of miR-148a in the oncogenic pathways of gastric, liver, breast and urogenital cancers, and in neurogliocytoma oncogenesis has been reported. Studying the functional role of miR-148a is crucial in discovering novel tumor molecular markers and identifying potential therapeutic targets 12. Role of miR-29b-1 has not been reported yet, but our results found that it is obviously correlated with OS and upregulated in LUAD patients. Consistent with our study, upregulation of miR-29b-1 has also been reported both in head and neck cancer and bladder urothelial carcinoma 13, 14. However, role and function of miR-29b-1 in cancer is still needed to explore. Multiple studies reported that miR-584 can function as a tumor suppressor by targeting different target genes, including WW domain-containing E3 ubiquitin protein ligase 1^15^, ROCK1^16^, MTDH^17^, PTTG1IP^18^, MMP-14 19 and so on. In this study, we found that miR-584 was downregulated in LUAD tissues. However, high expression of miR-584 is associated with worse OS in LUAD patients. Thus, our results indicated that it may also function as a potential oncogene in LUAD patients. Role of miR-4746 has not been reported yet, but our results found that it is the most correlated with OS in LUSC patients. And its predicted target gene is PIGA, which may involve in regulation of glycosylphosphatidylinositol (GPI)-anchor biosynthesis and metabolic pathways. Thus, further detection of miR-4746 in LUSC could be of great importance. Several studies have reported that miR-326 can inhibit cells proliferation, migration and invasion in various cancer types by targeting KRAS 20, TWIST1^21^, LIM, SH3 protein 1 22, ELK1 23 etc. However, in our study, we showed that high expression of miR-326 is associated with worse OS in LUSC patients. There are studies reported that miR-93 can promote different cancer cells proliferation, migration and progression by targeting PPARGC1A 24, PTEN 25, RB1 26, TIMP2 27, and so on. However, our results indicated that higher expression of miR-93 is correlated with better OS in LUSC patients. Yu and colleagues found that miR-671 was significantly upregulated in human prostate cancer tissues and cells. miR-671 overexpression promoted prostate cancer cell proliferation, while its downregulation inhibited prostate cancer cell proliferation. miR-671 directly targets the 3' untranslated region (UTR) of the tumor suppressor SOX6 (encoding SRY (sex determining region Y)-box 6) to inhibit its expression. Double knockdown of miR-671 and SOX6 promoted PC3 cell proliferation, suggesting that miR-671 promotes prostate cancer cell proliferation by inhibiting SOX6 28. In our study, we also found that miR-671 was upregulated in LUSC tissues, but the results indicated that higher expression of miR-671 is correlated with better OS in LUSC patients. Thus, the different roles of miRNAs in tumorigenesis might depend on tumor types and its target genes in different cells.

In this study, we found that in LUAD cases, miR-375, miR-148a, miR-29b-1 and miR-584 were differentially expressed between cancer samples and normal controls, and significantly associated with OS, while in LUSC cases, miR-4746, miR-326, miR-93 and miR-671 were differentially expressed between cancer samples and normal controls, and also significantly associated with OS. To obtain a deep insight into the molecular functions of these eight miRNAs, we predicted the target genes and analyzed the related pathways and GO annotations. Abnormal signaling pathways play crucial roles in the pathogenesis and progression of lung cancer. We found that the eight miRNAs could regulate several critical signaling pathways, including PI3K-Akt, TGF-beta, FoxO, Ras, GPI-anchor biosynthesis and metabolic, Rap1, HIF-1 and proteasome. Multiple evidence has demonstrated that all these pathways are differently involved in lung cancer development and progression 29-37. In the present study, we interestingly found that miR-375, miR-148a, miR-29b-1, miR-4746, miR-93 and miR-671 are upregulated in lung cancer samples compared with their normal controls. However, high expression of these miRNAs has better overall survival rates than low expression groups. Meanwhile, miR-584 and miR-326 were found downregulated in lung cancer samples. However, high expression of these miRNAs has worse overall survival rates than low expression groups. The possible explanations are as follows: The expression levels and functions of the miRNAs are changing in different stages of cancer, such as high expression associated with cancer development, and low expression associated with cancer progression. As miRNAs could function either as oncogenes or tumor suppressors by regulating different target genes, the miRNAs may have different target genes in different stages of cancer. A single miRNA could function exactly as an oncogene or tumor suppressor, while this effect could be inhibited by other equitable remedies. Thus, further molecular investigations are needed to confirm these predictions, and it can provide new therapeutic interventions in NSCLC.

Overall, eight miRNAs were found to be closely correlated with survival of NSCLC patients, and the constructed two four-miRNA signatures can be respectively used as potential prognostic predictors for LUAD and LUSC patients. Further studies are needed to validate our findings in large sample size, and further function investigation are also required to explore the molecular mechanism of these miRNAs in NSCLC development and progression.

## Conclusion

In this study, we identify the differentially expressed miRNAs (DEMs) between NSCLC and normal lung tissues, and evaluate the prognostic value and potential target gene functional enrichment of the DEMs. Moreover, we separately constructed two four-miRNA signatures (miR-375, miR-148a, miR-29b-1 and miR-584 for LUAD; miR-4746, miR-326, miR-93 and miR-671 for LUSC) that could effectively predict LUAD and LUSC patients' survival. Overall, eight miRNAs were closely correlated with survival of NSCLC patients, and the constructed two four-miRNA signatures could be respectively used as prognostic markers in LUAD and LUSC patients.

## Supplementary Material

Supplementary figures and tables.

## Figures and Tables

**Table 1:** Clinical characteristics of NSCLC patients.

Variables	LUAD case, n(%)	LUSC case, n(%)
Age at diagnosis		
<60	136(26.5%)	87(18.2%)
≥60	358(69.8%)	383(80.1%)
NA	19(3.7%)	8(1.7%)
T stage		
T1+T2	444(86.5%)	387(81.0%)
T3+T4	66(12.9%)	91(19.0%)
TX	3(0.6%)	
Lymph node status		
N0	330	301(63.0%)
N1-3	171	171(35.8%)
NX	11	6(1.2%)
NA	1	
Metastasis		
M0	346(67.4%)	390(81.6%)
M1	23(4.5%)	6(1.3%)
MX	140(27.3%)	78(16.3%)
NA	4(0.8%)	4(0.8%)
Stage		
I+II	398(77.6%)	388(81.2%)
III+IV	108(21.0%)	86(18.0%)
NA	7(1.4%)	4(0.8%)
Smoking history category		
<3	195(38.0%)	146(30.5%)
≥3	304(59.3%)	321(67.2%)
NA	14(2.7%)	11(2.3%)

NA, not available.

**Table 2:** The association between prognostic miRNAs and clinical features.

LUAD									
Variables	Numbers	hsa-mir-375	*P* value	hsa-mir-148a	*P* value	hsa-mir-29b-1	*P* value	hsa-mir-584	*P* value
Age at diagnosis									
<60	136	13.99±1.99	0.891	15.26±1.03	0.229	8.63±0.98	0.175	4.54±1.38	0.810
≥60	358	14.01±1.77		15.39±1.03		8.77±1.01		4.57±1.23	
T stage									
T1+T2	444	14.05±1.81	0.579	15.39±1.00	0.068	8.74±0.99	0.600	4.51±1.25	0.008
T3+T4	66	9.32±0.77		15.15±1.15		8.67±1.04		4.95±1.27	
Lymph node status									
N0	330	14.06±1.87	0.442	15.37±0.98	0.582	8.81±0.98	0.016	4.52±1.27	0.404
N1-3	171	13.93±1.78		15.31±1.10		8.58±1.02		4.62±1.25	
Metastasis									
M0	346	14.00±1.76	0.168	15.42±1.03	0.174	8.73±1.01	0.956	4.58±1.24	0.420
M1	23	14.54±2.51		15.12±1.15		8.72±1.11		4.80±1.57	
Stage									
I+II	398	14.02±1.78	0.722	15.39±0.99	0.086	8.79±0.98	0.011	4.51±1.23	0.026
III+IV	108	13.95±1.99		15.20±1.12		8.52±1.04		4.81±1.34	
Smoking history category									
<3	195	14.05±1.99	0.819	15.49±1.01	0.018	8.68±0.93	0.315	4.61±1.28	0.607
≥3	304	14.02±1.74		15.27±1.02		8.77±1.03		4.55±1.27	
**LUSC**									
**Variables**	**Numbers**	**hsa-mir-4746**	***P* value**	**hsa-mir-326**	***P* value**	**hsa-mir-93**	***P* value**	**hsa-mir-671**	***P* value**
Age at diagnosis									
<60	87	2.71±0.96	<0.001	2.39±1.21	0.887	13.20±1.03	0.001	4.12±0.66	<0.001
≥60	383	2.27±0.96		2.41±1.13		12.79±0.98		3.70±0.66	
T stage									
T1+T2	387	2.34±0.98	0.929	2.44±1.16	0.657	12.87±0.99	0.673	3.79±0.66	0.639
T3+T4	91	2.35±0.93		2.38±1.13		12.82±1.03		3.75±0.73	
Lymph node status									
N0	301	2.31±0.96	0.194	2.44±1.11	0.618	12.83±0.95	0.336	3.76±0.67	0.304
N1-3	171	2.43±0.99		2.38±1.21		12.93±1.08		3.83±0.68	
Metastasis									
M0	390	2.37±0.98	0.055	2.38±1.11	0.246	12.86±0.99	0.031	3.78±0.67	0.366
M1	6	1.59±0.55		1.85±1.18		11.98±0.68		3.53±0.41	
Stage									
I+II	388	2.36±0.99	0.546	2.44±1.13	0.420	12.88±0.98	0.673	3.77±0.67	0.520
III+IV	86	2.29±0.92		2.33±1.24		12.83±1.10		3.82±0.71	
Smoking history category									
<3	146	2.41±0.88	0.224	2.55±1.10	0.120	13.01±0.90	0.018	3.83±0.67	0.232
≥3	321	2.30±1.01		2.37±1.16		12.78±1.01		3.75±0.67	

**Table 3:** Univariate and multivariate Cox regression analysis in NSCLC patients.

	LUAD				LUSC			
	Univariate analysis		Multivariate analysis		Univariate analysis		Multivariate analysis	
	HR (95% CI)	*P* value	HR (95% CI)	*P* value	HR (95% CI)	*P* value	HR (95% CI)	*P* value
Age (≥60 vs. <60)	1.06(0.71-1.57)	7.91E-01	1.37(0.83-2.27)	2.19E-01	1.11(0.69-1.79)	6.54E-01	1.46(0.81-2.63)	2.06E-01
Metastasis (M1 vs. M0)	1.73(0.92-3.25)	8.25E-02	0.82(0.39-1.73)	5.95E-01	2.04(0.64-6.47)	2.17E-01	0.88(0.21-3.77)	8.65E-01
Lymph node status (N1-3 vs. N0)	2.58(1.80-3.69)	**9.22E-08**	1.63(0.97-2.74)	6.61E-02	1.21(0.87-1.69)	2.62E-01	0.96(0.62-1.49)	8.49E-01
Clinical stage (III+IV vs. I+II)	2.90(2.01-4.186)	**2.54E-09**	1.77(0.98-3.20)	5.77E-02	1.54(1.04-2.27)	**2.89E-02**	1.19(0.68-2.07)	5.50E-01
T stage (T3+T4 vs. T1+T2)	2.47(1.54-3.95)	**1.73E-04**	2.22(1.21-4.07)	**1.03E-02**	1.53(1.05-2.21)	**2.49E-02**	1.38(0.76-2.50)	2.90E-01
Smoking history category (≥3 vs. <3)	1.15(0.78-1.69)	4.82E-01	1.42(0.90-2.26)	1.33E-01	0.76(0.53-1.07)	1.16E-01	0.59(0.39-0.90)	**1.33E-02**
Four miRNA signature (high risk vs. low risk)	1.85(1.29-2.65)	**6.76E-04**	1.78(1.15-2.74)	**9.66E-03**	1.85(1.33-2.57)	**2.32E-04**	2.44(1.66-3.59)	**5.17E-06**

## Abbreviations

MiRNAs: microRNAs
DEMs: differentially expressed miRNAs
NSCLC: non-small cell lung cancer
TCGA: The Cancer Genome Atlas Project
LUAD: lung adenocarcinoma
LUSC: lung squamous cell carcinoma
FCs: fold changes
OS: overall survival
DAVID: The Database for Annotation, Visualization and Integrated Discovery
GO: Gene Ontology
KEGG: Kyoto Encyclopedia of Genes and Genomes
PPI: protein-protein interaction
STRING: Search Tool for the Retrieval of Interacting Gene
SD: standard deviation
BP: biological process
CC: cellular component
MF: molecular function
UTRs: untranslated regions
